# Acne located on the trunk, whey protein supplementation: Is there any association?

**DOI:** 10.15171/hpp.2017.19

**Published:** 2017-03-05

**Authors:** Fatma Pelin Cengiz, Bengu Cevirgen Cemil, Nazan Emiroglu, Anil Gulsel Bahali, Nahide Onsun

**Affiliations:** ^1^Department of Dermatoveneorology, Bezmialem Vakif University, Istanbul, Turkey; ^2^Department of Dermatoveneorology, Diskapi Yildirim Beyazit Training and Research Hospital, Ankara, Turkey

**Keywords:** Acne, Adolescent, Protein supplement

## Abstract

Whey protein is a source of protein that was isolated from milk. Whey proteins are composed of higher levels of essential amino acids. The role of diet in acne etiology has been investigated for several years. It was established that milk and milk products can trigger acneiform lesions, and recent evidence supports the role of whey protein supplements in acne. Herein, we report 6 healthy male adolescent patients developing acne located only to the trunk after the consumption of whey protein supplements for faster bodybuilding. This is the first observation which specified the location of acneiform lesions among bodybuilders. In our opinion, a trendy and common health problem is beginning among adolescents in the gyms.

## Introduction

Acne vulgaris is the chronic inflammatory disease of the pilosebaceous unit with multifactorial pathogenesis involving genetic factors, hormone imbalance, increased sebum production, abnormal keratinization, and bacterial proliferation. The disease is characterized by seborrhea and clinical presentation with comedones, pustules and papules. It was suggested in the literature that milk consumption may be a cause for acne outbreaks. Although milk has a low glycemic index, it could aggravate acne by increasing the levels of insulin like growth factor-1 (IGF-1) as well as releasing comedogenic hormones such as estrogen, progesterone, androgen precursors and 5a-reductase steroids.^1,2^ Whey protein is a mixture of globular proteins isolated from whey, the liquid material constituted as a product of cheese. Whey protein is sold as a dietary supplement, especially bodybuilders believe that they can improve performance and gain muscle mass when consuming whey protein supplements. Adolescent use of protein supplements is becoming a common health problem. Herein, we report 6 cases of acne lesions located only to the trunk in patients who use protein supplements for bodybuilding.

## Case Report

Retrospective analysis of 6 consecutive patients with acne located on trunk associated with protein supplement intake, seen between February 2016 and June 2016. The history, clinical charts, laboratory tests, clinical evaluation, were reviewed.

Patients included in the study were all men, with a mean age of 18 years (range 16-18). They used protein supplement to improve their performance and gain muscle mass. They took supplement throughout the day. All of them remarked that their lesions began after the usage of protein supplements (mean: 3.1 ± 1.7 month). None of them reported the use of anabolic steroids, drugs, alcohol consumption or smoking (Table 1).

**Figure 1:**
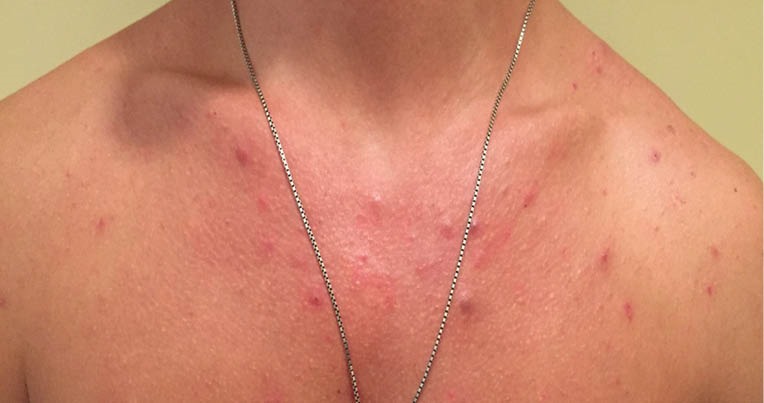
Acne located on presternal region.

**Figure 2:**
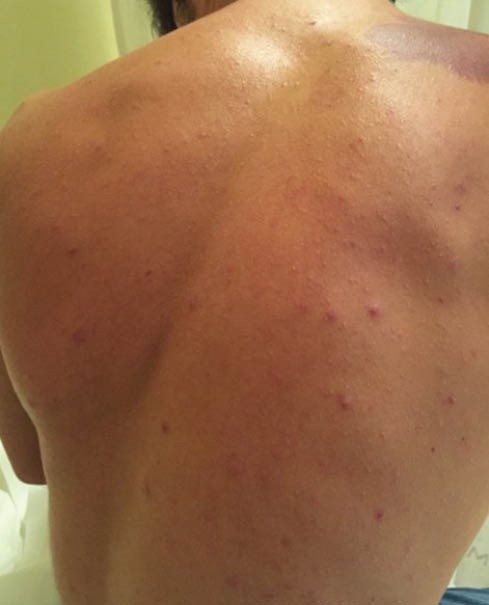
Acne located on the back.

In all our cases, a normal hemogram was obtained during further examination. The levels of serum electrolytes, blood sugar, creatinine, alanine transaminase (ALT), aspartate aminotransferase (AST), alkaline phosphatase (ALP) and total bilirubin were also normal. We, therefore, checked for anti-HAV IgM, HBsAg, anti-HBc IgM, and anti-HCV antibodies, but the results were negative. On examination, they had papulonodular acne on their chest and back without involvement of face (Figures 1 and 2). We prescribed oral tetracyline and clindamycin- benzoyl peroxide gel. The patients experienced mild-moderate improvement of their acne after discontinuation of protein supplement and administration of tetracyline and clindamycin- benzoyl peroxide gel. We had associated the improvement of acneiform lesions with discontinuation of whey protein more than treatment regimens for acne, since two of our patients preferred to not discontinue their whey protein supplementation firstly, and they had less improvement than others.

## Discussion

Protein supplements, particularly whey protein, are preferred by young people and adolescents for faster muscle building, with no follow-up. Whey is left over when milk is coagulated during the process of cheese production, and it is composed of beta-lactoglobulin (~65%), alpha-lactalbumin (~25%), bovine serum albumin, and immunoglobulins.

It has been established that high glycemic load (HGL) diets, high intakes of carbonhydrates, milk consumption may trigger acne outbreaks. The milk can increase the levels of insulin like growth factor-1 (IGF-1), and IGF can induce keratinocyte proliferation and apoptosis.^2^ In addition to increased expressions of insulin/IGF-1 receptors in epidermal keratinocytes, IGF-1 also stimulates 5α-reductase, adrenal and gonadal androgen synthesis, androgen receptor signal transduction.^3^ It was demonstrated that protein supplements as well as milk can induce acneiform lesions.^4^ The nutritional supplements which bodybuilders prefer to use, have the same amount of whey protein with 6-12 liters of milk as concentrated formulas.

Pontes et al showed that at the beginning of their follow-up, without protein supplementation, only 56.7% of their patients had been presented acneiform lesions, with degrees that varied from I to II. After 2 months using the protein-calorie supplement, all of the patients had acneiform lesions, 30% of them with degree III.^5^ They also observed that increase in acneiform lesions was more significant during the first month of supplement use than during the second. They suggested that acneiform lesions decreases by time.

The findings of Simonart were similar to ours. His patients had developed acne outbreak after initiation of whey protein supplements, especially on the face and trunk. The patient who had discontinued supplement, had the best cosmetic result with acne treatment.^6^

## Conclusion

In our case series, our patients determined the outbreak of acneiform lesions after their beginning of protein supplements, and we observed that their lesions were located only to the trunk, not to face. It may be associated with increased friction, perspiration on the back. This association between location of acneiform lesions and protein supplements should be supported by larger studies.

## Ethical approval

Written informed consent was taken from all patients.

## Competing interests

The authors declare no conflict of interest.

## Authors’ contributions

Concept - FPC; Design - BBC; Supervision - NO; Materials - NE; Data collection and/or processing - AGB; Literature review - BCC; Writing - FPC; Critical review - NO.

**Table 1:** Clinical features of patients

**Age (y)**	**BMI**	**Frequency of whey protein consumption/week**	**Global acne grading system**
16	26	3	20
18	25	4	24
18	23	3	18
17	23	3	22
17	25	7	28
16	24	3	25
